# Clinical Efficacy of Vitamin D Supplements for the Treatment of Anemia in Patients With Chronic Kidney Disease: A Systematic Review and Meta‐Analysis

**DOI:** 10.1111/hdi.70078

**Published:** 2026-06-07

**Authors:** Xiaodong Wanyan, Jiayun Xu

**Affiliations:** ^1^ Department of Nephrology The First Affiliated Hospital of Henan University of Science and Technology Luoyang China

**Keywords:** anemia, chronic kidney disease, meta‐analysis, vitamin D

## Abstract

**Objective:**

To conduct a meta‐analysis of clinical randomized controlled studies related to vitamin D supplements for the treatment of anemia in patients with chronic kidney disease (CKD), to evaluate the clinical efficacy of vitamin D supplements for CKD patients complicated with anemia.

**Methods:**

A literature review of the published literature in both English and Chinese was searched from the Cochrane Library, PubMed, Web of Science, CNKI and Wanfang Data that were published from the inception date to January 2018. Randomized controlled trials (RCTs) eligible for the inclusion and exclusion criteria were selected. The quality of eligible studies was assessed by the Cochrane Risk of Bias tool. Meta‐analysis was performed by using Revman 5.3 software.

**Results:**

A total of 12 RCTs comprising 779 patients were included in this meta‐analysis, including 383 patients in the experimental group (with vitamin D supplementation) and 396 patients in the control group (without vitamin D supplementation). Meta‐analysis showed that vitamin D supplements could significantly increase the hemoglobin (*p* < 0.01, SMD = 1.24, 95% CI: 1.02–1.45) and lower the dosage of erythropoietin required (*p* < 0.01, SMD = −0.76, 95% CI: −1.09 to −0.43).

**Conclusion:**

Use of vitamin D supplements can significantly elevate the hemoglobin concentration and reduce the dosage of erythropoietin. However, these findings remain to be validated by large‐scale, multi‐center, prospective RCTs.

## 1 Introduction

Patients with chronic kidney disease (CKD), who have a decline of kidney function, may have a decrease in the production of active vitamin D, thereby leading to vitamin D deficiency [1]. Vitamin D has been described to have multiple biological effects, such as direct suppression of hepcidin, anti‐inflammatory and immune‐modulatory effects, direct stimulation of erythropoiesis in the bone marrow, potential support for endogenous erythropoietin production, and reduction of parathyroid hormone, etc. [2, 3]. Consequently, vitamin D can not only maintain the balance between calcium and phosphorus metabolism, regulate bone metabolism, and inhibit parathyroid hormone secretion, but also improve the hemoglobin level or reduce the dosage of erythropoietin administration needed in CKD patients. Patel et al. [4] conducted a multi‐center cohort study consisting of 1661 CKD patients from the United States and found that the deficiency of 25(OH)D and 1,25(OH)D levels was significantly associated with the decrease of hemoglobin level in CKD patients. Vitamin D supplements have also been proven to alleviate the anemia status in patients diagnosed with CKD [5, 6, 7, 8, 9] probably through multiple effects.

In this meta‐analysis, all randomized controlled trials (RCTs) related to clinical efficacy of vitamin D supplements in the treatment of anemia in CKD patients were searched to provide evidence‐based data for basic research and clinical treatment decision‐making for the interventions in CKD patients who have anemia.

## 2 Materials and Methods

### 2.1 Literature Searching Strategy

A literature review was conducted using the keywords of “vitamin D or calcitriol or paricalcitol or cholecalciferol or ergocalciferol,” and “hemoglobin or anemia,” and “chronic kidney disease or kidney insufficiency or chronic kidney failure or kidney failure” and “anemia” in both English and Chinese from EMBASE, the Cochrane Library, PubMed, Web of Science, CNKI and Wanfang Data published from the inception date to January 2018. All retrieval strategies were determined according to the Population, Intervention, Comparison, Outcomes and Study designs (PICOS) principle.

### 2.2 Inclusion and Exclusion Criteria

Inclusion criteria: published studies related to the effect of vitamin D supplementation on anemia in CKD patients in both Chinese and English were searched. Exclusion criteria: Review articles, case reports, abstracts and basic animal experiments; Articles related to patients with vitamin D concentration of > 30 ng/mL and anemia; patients complicated with other chronic diseases which may lead to a decrease in hemoglobin level, such as patients with malignant tumors, congestive heart failure, blood system diseases and immune system diseases, etc.; Multiple studies carried out in the same or overlapping populations, not all studies were included, only the most relevant and detailed studies were selected; Studies lacking original data or relevant data required for meta‐analysis; Non‐RCTs, observational and cross‐sectional studies were also excluded from this meta‐analysis.

### 2.3 Data Screening and Extraction

Two independent investigators were responsible for examining the title, abstract, and full text, and cross‐checked and screened the research type of the literature, clinical characteristics of the enrolled patients, intervention measures, and the previously determined inclusion and exclusion criteria. If these two investigators had disagreement regarding one certain article, they should settle the discrepancy through consultation or invite a third party to determine whether this article should be included or not. The extracted data mainly included the first author's name, publication time, nationality, baseline data of the enrolled patients, sample size, intervention and controlled measures, and course of treatment. Hemoglobin concentration and dose of erythropoietin were the main observational parameters.

### 2.4 Literature Quality Assessment

The internal effectiveness of all RCTs that met the inclusion criteria and did not meet the exclusion criteria was evaluated by the Cochrane Risk of Bias tool. The quality assessment of the included RCTs was performed by using the Cochrane Risk of Bias tool according to the following criteria: random sequence generation, allocation concealment, blinding of participants and personnel, blinding of outcome assessment, incomplete outcome data (loss to follow‐up), selective reporting, and other bias. Studies with a low risk of bias in three domains were categorized as having low overall risk of bias. All other studies were classified as having a high overall risk of bias.

### 2.5 Statistical Analysis

Statistical analysis was carried out by using Review Manager 5.3 software. The heterogeneity test was conducted by using the *χ* ^2^ test. The size of heterogeneity was expressed by *I* ^2^. *I* ^2^ < 50% indicates that the heterogeneity among different groups is within an acceptable range, and the possibility of homogeneity is considered. A fixed effect model was used for the analysis of homogeneous data. *I* ^2^ > 50% suggests that the homogeneity among different groups is small and the degree of heterogeneity is high. The random effect model was adopted for data analysis with heterogeneity. Odds ratio (OR) and risk ratio (RR) were utilized as the effect indicators for the binary variables. For continuous variables, mean differences (MD) or standardized mean differences (SMD) were used as the effect indicators, and 95% confidence interval (95% CI) was calculated for the results, and *p* < 0.05 was defined as the boundary. The Vitamin D group was the experimental group, and the placebo group was the control group. The publication bias was assessed by the funnel plot. Symmetrical two sides of the funnel plot indicated that there was no significant publication bias, whereas if the two sides were asymmetrical, publication bias may be induced.

## 3 Results

### 3.1 Literature Researching Outcomes

**FIGURE 1:**
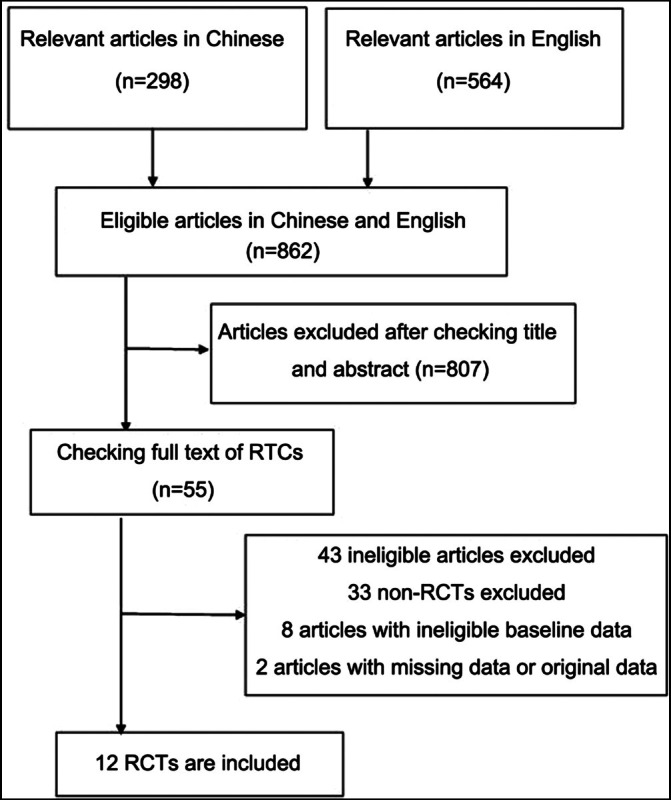
Study screening flow chart.

As described in Figure 1, a total of 862 related studies were retrieved, including 298 articles in Chinese and 564 in English. According to the inclusion and exclusion criteria, the retrieved articles were subsequently screened. Finally, 55 articles were obtained by examining the titles and abstracts to eliminate duplicate studies, animal experiments, in vitro experiments, and case reports, etc. Then, by examining the full text, 43 articles that did not meet the inclusion criteria were excluded. Among them, 33 articles were considered as non‐RCTs. In eight studies, the initial vitamin D dose was > 30 ng/mL or the observational parameters were inconsistent with the requirements in this meta‐analysis. Two studies with incomplete data or lacking the original data were excluded. Finally, 12 eligible RCTs consisting of 779 patients were included in this meta‐analysis, including 383 patients in the experimental group (with vitamin D supplementation) and 396 patients in the control group (without vitamin D supplementation). No statistical differences were noted in baseline data between the two groups (all *p* > 0.05), as illustrated in Table 1.

**TABLE 1:** Baseline information of the included RCTs.

Citation	Year	Country	Sample size	Course of treatment	Parameter
A.E. et al. [10]	2015	Iran	64	4 months	VitD, Hb
D.C. et al. [11]	2015	US	252	6 months	EPO, Fer, PTH, CRP, Ca
E.R. et al. [12]	2015	Italy	60	6 months	VitD, Hb, Fer, PTH, CRP, Ca
F.H. et al. [13]	2014	Denmark	50	6 months	VitD, Hb, PTH, CRP, Ca
B.V. et al. [14]	2016	Italy	34	6 months	Hb
P.R. et al. [15]	2013	Thailand	20	3 months	VitD, Hb, Fer, PTH, CRP, Ca
M.A. et al. [16]	2016	US	29	4 months	CRP
Y.N. et al. [17]	2012	Egypt	60	6 months	Hb, EPO
B.P. et al. [18]	2018	US	40	6 weeks	Fer, TS
Yuan et al. [19]	2015	China	50	4 months	Hb, PTH, Ca, P
Yang et al. [20]	2001	China	40	10 weeks	Hb, PTH, Ca, P
Wu et al. [21]	2014	China	80	6 months	Hb, EPO, PTH, Ca, P, Fer

### 3.2 Risk Bias Assessment Outcomes

**FIGURE 2:**
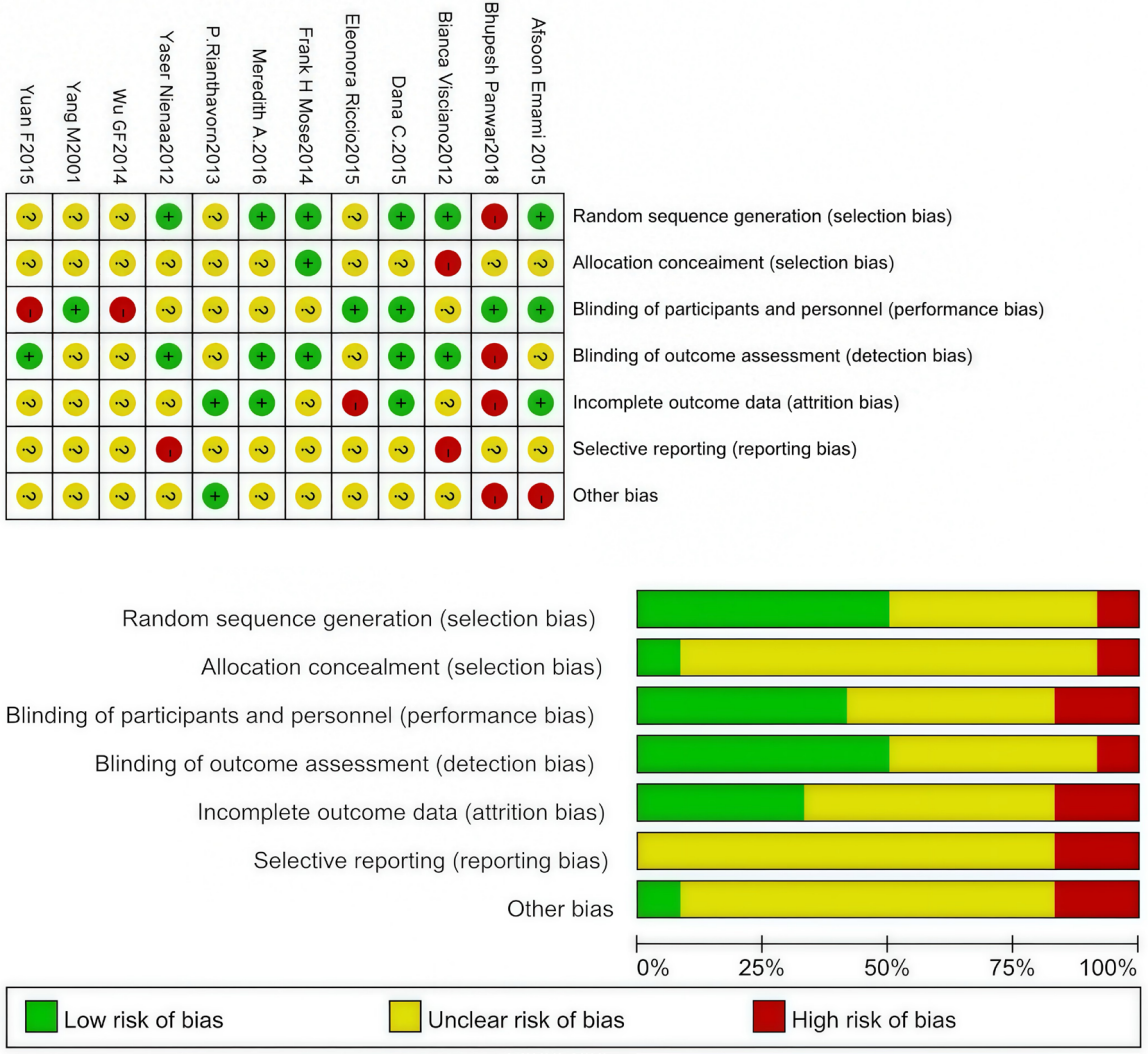
Risk bias assessment.

Studies with a low risk of bias in three domains were categorized as having a low overall risk of bias. All other studies were classified as having a high overall risk of bias. According to the assessment criteria of the Cochrane Risk of Bias tool, two studies (A.E et al. and D.C et al.) were categorized as having a low risk of bias due to a low risk of bias in three domains. The remaining 10 RCTs were classified as having a high risk of bias, as demonstrated in Table 2 and Figure 2.

**TABLE 2:** Literature quality assessment outcomes of included 12 RCTs.

Citation	Random sequence generation	Allocation concealment	Blinding of participants and personnel	Blinding of outcome assessment	Incomplete outcome data	Selective reporting	Other bias
A.E. et al. [10]	Low	Unclear	Low	Unclear	Low	Unclear	Unclear
D.C. et al. [11]	Unclear	Unclear	Low	Low	Low	Unclear	Unclear
E.R. et al. [12]	Unclear	Unclear	Low	Unclear	High	Unclear	Unclear
F.H. et al. [13]	Unclear	Low	Unclear	Low	High	Unclear	Unclear
B.V. et al. [14]	Unclear	High	Unclear	Low	High	Unclear	High
P.R. et al. [15]	Unclear	Unclear	Unclear	High	Low	Unclear	Low
M.A. et al. [16]	High	Unclear	Unclear	Low	Low	Unclear	High
Y.N. et al. [17]	Low	Unclear	Unclear	Low	Unclear	High	Unclear
B.P. et al. [18]	High	Unclear	Low	High	High	Unclear	Unclear
Yuan et al. [19]	Unclear	Unclear	High	High	Unclear	High	High
Yang et al. [20]	Unclear	Unclear	Low	High	Unclear	Unclear	Unclear
Wu et al. [21]	Unclear	Unclear	High	Unclear	Unclear	Unclear	Unclear

### 3.3 Effect of Vitamin D Supplements on 25(OH)D Concentration

**FIGURE 3:**
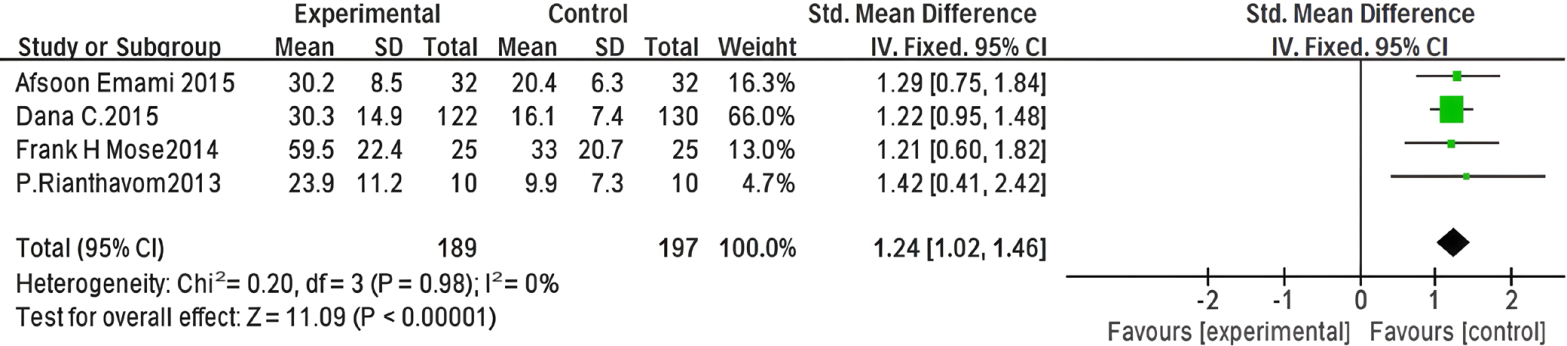
Forest plot of effect of vitamin D supplements on 25(OH)D concentration in CKD patients.

In four RCTs, the effect of vitamin D supplementation on serum 25(OH)D concentration in patients with CKD was evaluated. A total of 386 patients were enrolled, including 189 cases in the experimental group and 297 in the control group. As shown in Figure 3, there was no heterogeneity among different studies (all *p* < 0.01, *I* ^2^ = 0%). The fixed effect model was adopted. The results of meta‐analysis indicated that there was a significant difference in the serum 25(OH)D concentration between the experimental and control groups (*p* < 0.01, SMD = 1.24, 95% CI: 1.02–1.46). These results revealed that vitamin D supplementation significantly increased serum 25(OH)D levels and effectively mitigated vitamin D deficiency in CKD patients.

### 3.4 Effect of Vitamin D Supplements on Hemoglobin Concentration

**FIGURE 4:**
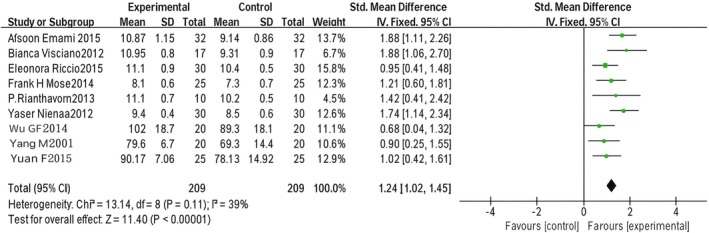
Forest plot of effect of vitamin D supplements on hemoglobin concentration in CKD patients.

The effect of vitamin D supplementation on the hemoglobin levels in CKD patients complicated with anemia was investigated in nine RCTs comprised of 418 patients. Among them, 209 patients were allocated into the experimental group and 209 cases in the control group, respectively. As shown in Figure 4, the heterogeneity test indicated significant heterogeneity among different studies (*p* < 0.01, *I* ^2^ = 39%), which were still within the acceptable range. The fixed effect model was adopted. After the adjustment for multiple factors, the heterogeneity was maintained within the acceptable range. Subsequently, meta‐analysis results showed that there was a significant difference in the hemoglobin levels between the experimental and control groups (*p* < 0.01, SMD = 1.24, 95% CI: 1.02–1.45). These findings suggested that vitamin D supplementation increased the hemoglobin concentration and alleviated anemia in CKD patients complicated with anemia.

### 3.5 Effect of Vitamin D Supplements on the Dosage of Erythropoietin

**FIGURE 5:**
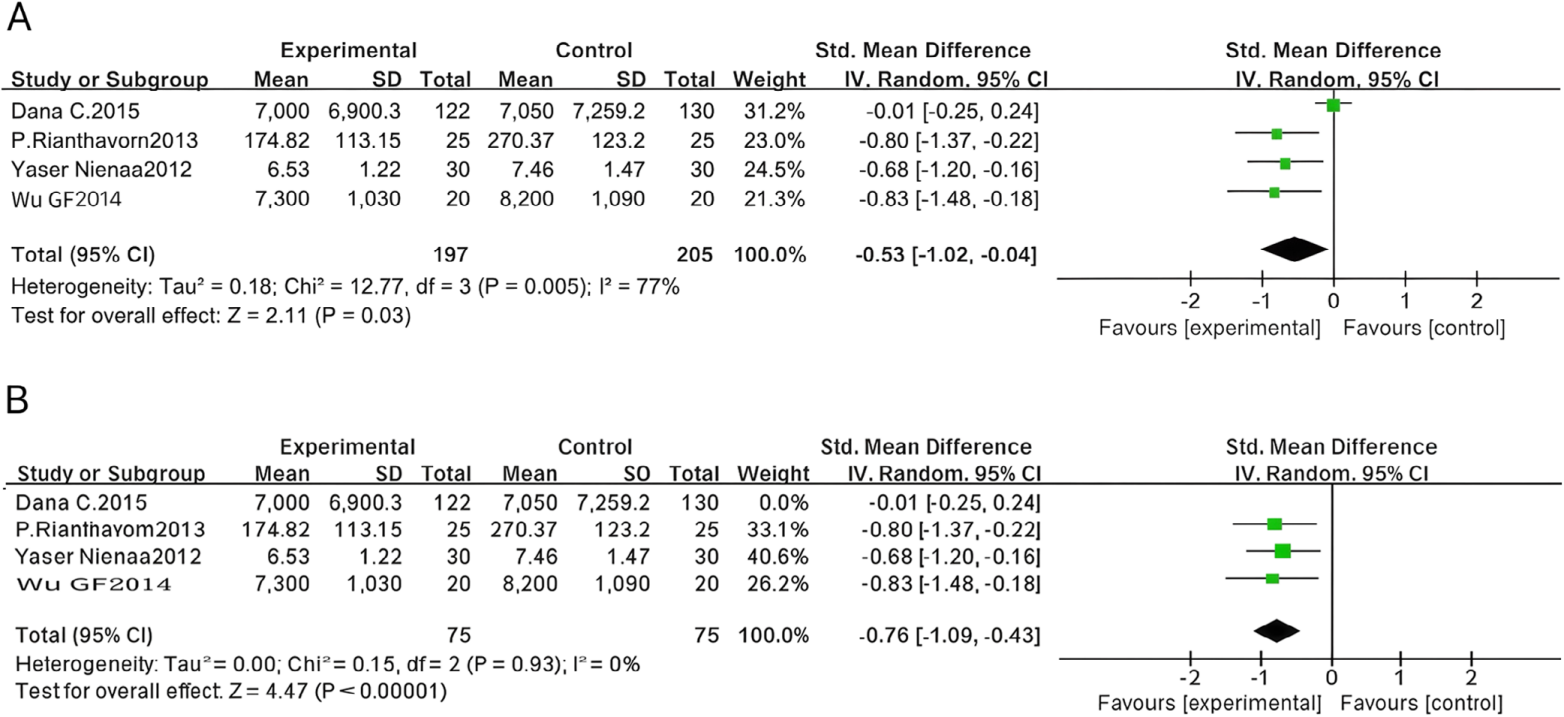
Forest plot of effect of vitamin D supplements on the dosage of erythropoietin in CKD patients before (A) and after heterogeneity test (B).

As shown in Figure 5A, there was significant heterogeneity among different studies (*p* = 0.03, *I* ^2^ = 77%); therefore, the results might be unreliable if meta‐analysis was conducted without the factor adjustment. After the adjustment for confounding factors, significant heterogeneity was observed in the study of Dana et al., and this study was excluded. Then, the heterogeneity test was repeated, and there was no heterogeneity among different studies (*p* < 0.01, *I* ^2^ = 0%), as illustrated in Figure 5B. In four studies, the changes of erythropoietin dosage between the experimental and control groups after vitamin D supplementation were statistically compared. Among 402 patients, 197 cases were assigned to the experimental group and 205 cases to the control group, respectively. Using the fixed effect model, meta‐analysis results showed that the dosage of erythropoiesis in the experimental group was significantly lower than that in the control group after vitamin D supplementation (*p* < 0.01, SMD = −0.76, 95% CI: −1.09 to −0.43). However, considering the large sample size of the study by Dana et al., the sample size of the remaining three studies was too small, and the results of meta‐analysis were not completely reliable with limited reference value.

### 3.6 Publication Bias and Sensitivity Analysis

**FIGURE 6:**
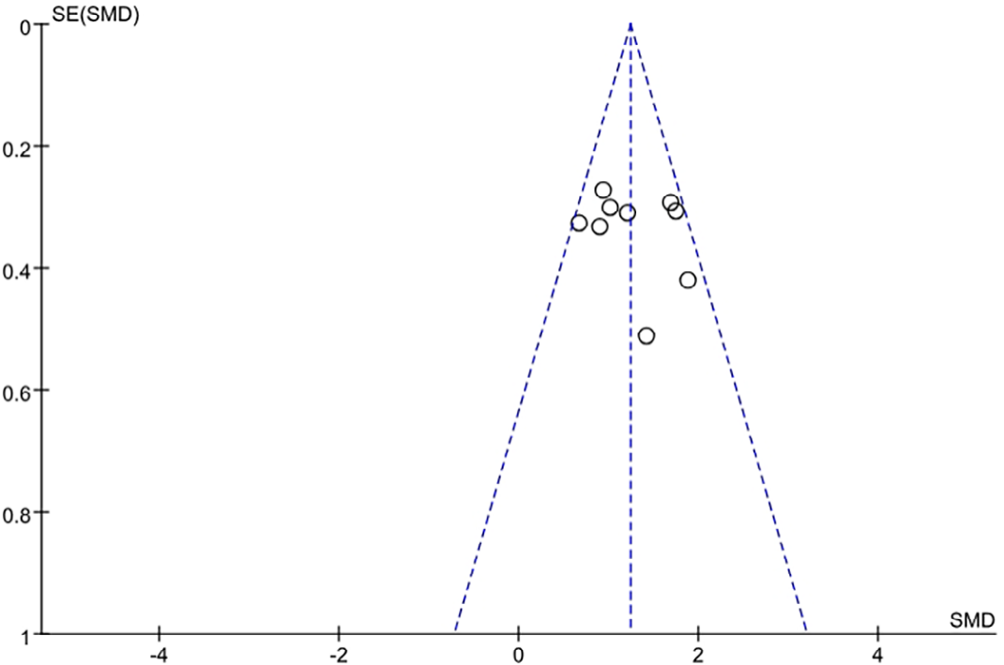
Funnel plot of the effect of vitamin D supplementation on hemoglobin levels in CKD patients complicated with renal anemia.

In this meta‐analysis, the publication bias was evaluated by using Review Manger 5.3 software. As shown in Figure 6, nine included articles were generally distributed surrounding the combined overall effect, but the scattering distribution was not completely symmetrical, suggesting that a certain degree of publication bias might exist. The data were merged for meta‐analysis, or the model was switched to eliminate a certain study, and then the statistical analysis was carried out. The obtained results were still consistent with the original meta‐analysis, indicating that the meta‐analysis results were highly reliable and possessed certain clinical reference value.

## 4 Discussion

CKD has become one of the most prevalent kidney diseases worldwide [1]. With growing incidence of diabetes mellitus and an aging population, the incidence of CKD has been increasingly elevated. CKD patients suffer from a variety of complications, such as volume overload, electrolyte imbalances, disordered mineral and bone metabolism, and anemia [1, 22]. Although the main causes of CKD‐induced anemia include erythropoietin deficiency, iron deficiency, and malnutrition, recent studies suggest deficiencies in vitamin D may act as an additional pathophysiological factor of anemia [23].

In one cross‐sectional study, Patel et al. [4] have observed a linear relationship between vitamin D and hemoglobin concentrations. Multivariable analysis revealed a stepwise decline in hemoglobin concentrations and a stepwise increase in the prevalence of anemia with decreasing tertiles of vitamin D concentrations. Moreover, dual deficiency of 25D and 1,25D exerts an additive effect upon the incidence of anemia. Each of these associations is independent of multiple potential confounding factors, suggesting that vitamin D deficiency is a potential risk factor for anemia in CKD. Sim et al. [5] have also observed similar findings that vitamin D deficiency is correlated with a higher risk of anemia, lower mean hemoglobin level, and higher usage dosage of erythrocyte‐stimulating agents, which are consistent with the outcomes obtained in the present meta‐analysis. There is unanimity in multiple studies [8, 9, 22, 23] indicating that vitamin D deficiency is directly associated with the risk of anemia induced by chronic diseases or inflammation in both adult and pediatric patients.

In this meta‐analysis, the latest RCTs focusing on the effect of vitamin D supplementation on anemia in CKD patients and vitamin D deficiency were summarized. In addition, the results of multiple studies were comprehensively analyzed, and the inconsistency of the results among different RCTs was assessed to increase the reliability of the conclusions. The results showed that compared with the placebo control group (CKD patients with vitamin D deficiency), the hemoglobin level of CKD patients was significantly elevated and clinical efficacy of corresponding interventions was also enhanced after vitamin D supplementation, which were consistent with the findings obtained by Ahmad et al. [24]. In our study, more RCTs were included for meta‐analysis, probably adding more evidence to clinical practice. In addition, the results of this meta‐analysis are consistent with the findings in previous studies that vitamin D supplementation can increase the 25(OH)D concentration of dialysis patients and enhance the sensitivity of erythropoietin, thereby reducing the body's demand for erythropoietin‐stimulating factors, and it can achieve equivalent efficacy in the management of anemia [15, 25, 26, 27, 28, 29, 30, 31]. Rattanasompattikul et al. [25] indicated that a decreased responsiveness to erythropoiesis‐stimulating agents is one of the major causes of the risk of refractory anemia. In their cohort study of 754 hemodialysis patients, malnutrition biomarkers, such as vitamin D deficiency and low hemoglobin level, are independent predictors of worse responsiveness to erythropoiesis‐stimulating agents in hemodialysis patients. Therefore, although the exact mechanism for the decline in the sensitivity of hematopoietic system to erythropoiesis‐stimulating factor remains elusive, preliminary findings that vitamin D supplementation can significantly improve the sensitivity of erythropoiesis‐stimulating agents in bone marrow hematopoietic system are of significant value [32].

Several limitations are acknowledged in this meta‐analysis. First, the quantity, quality, and sample size of RCTs included in this meta‐analysis are relatively low. High heterogeneity and errors in the random effect model affect the credibility of the conclusions. Moreover, vitamin D supplements in different studies were considered as the same type, and subgroup analysis was not conducted to analyze different types of vitamin D in this meta‐analysis. The findings remain to be validated by large sample size, multi‐center, and prospective RCTs.

## 5 Conclusions

Taken together, the use of vitamin D supplements and erythropoietin can significantly elevate the hemoglobin concentration and reduce the dosage of erythropoietin. However, the exact mechanism is elusive. These findings remain to be validated by large‐scale, multicenter, and prospective RCTs.

## Funding

The authors have nothing to report.

## Conflicts of Interest

The authors declare no conflicts of interest.

## Data Availability

The data that support the findings of this study are available from the corresponding author upon reasonable request.
